# Do cognitive aids reduce error rates in resuscitation team performance? Trial of emergency medicine protocols in simulation training (TEMPIST) in Australia

**DOI:** 10.1186/s12960-019-0441-x

**Published:** 2020-01-08

**Authors:** Charlotte Hall, Dean Robertson, Margaret Rolfe, Sharene Pascoe, Megan E. Passey, Sabrina Winona Pit

**Affiliations:** 1 University Centre for Rural Health, School of Medicine, Western Sydney University, 62 Uralba Street, Lismore, NSW 2480 Australia; 2 Maclean District Hospital, Maclean, NSW Australia; 3 School of Rural Health, The University of Sydney, University Centre for Rural Health, Lismore, Australia

**Keywords:** Simulation training, Emergency room, Emergency department, Cognitive aids, Checklists, Emergency crisis, Resuscitation, Crisis error rates, Guidelines, Team performance, Non-technical skills, Patient safety

## Abstract

**Background:**

Resuscitation of patients with time-critical and life-threatening illness represents a cognitive challenge for emergency room (ER) clinicians. We designed a cognitive aid, the Emergency Protocols Handbook, to simplify clinical management and team processes. Resuscitation guidelines were reformatted into simple, single step-by-step pathways. This Australian randomised controlled trial tested the effectiveness of this cognitive aid in a simulated ER environment by observing team error rates when current resuscitation guidelines were followed, with and without the handbook.

**Methods:**

Resuscitation teams were randomised to manage two scenarios with the handbook and two without in a high-fidelity simulation centre. Each scenario was video-recorded. The primary outcome measure was error rates (the number of errors made out of 15 key tasks per scenario). Key tasks varied by scenario. Each team completed four scenarios and was measured on 60 key tasks. Participants were surveyed regarding their perception of the usefulness of the handbook.

**Results:**

Twenty-one groups performed 84 ER crisis simulations. The unadjusted error rate in the handbook group was 18.8% (121/645) versus 38.9% (239/615) in the non-handbook group. There was a statistically significant reduction of 54.0% (95% CI 49.9–57.9) in the estimated percentage error rate when the handbook was available across all scenarios 17.9% (95% CI 14.4–22.0%) versus 38.9% (95% CI 34.2–43.9%). Almost all (97%) participants said they would want to use this cognitive aid during a real medical crisis situation.

**Conclusion:**

This trial showed that by following the step-by-step, linear pathways in the handbook, clinicians more than halved their teams’ rate of error, across four simulated medical crises. The handbook improves team performance and enables healthcare teams to reduce clinical error rates and thus reduce harm for patients.

**Trial registration:**

ACTRN12616001456448 registered: www.anzctr.org.au. Trial site: http://emergencyprotocols.org.au/

## Background

The management of life-threatening, time-critical emergencies is often challenging in any emergency room. A Canadian literature [1] review and a systematic review [2] without any language restrictions found that the resuscitation of a patient in extremis often occurs in chaotic circumstances with an incomplete clinical picture and the requirement for rapid decision-making [1, 2]. US [3] and German [4] human factors research has shown that during a crisis, memory worsens, cognition is overloaded, performance degrades, and distractions interrupt planned actions [3, 4]. High levels of stress and fatigue decrease cognitive functioning [5]. Emergency room clinicians work within such complex, high-stakes environments around the clock.

Australian emergency rooms are becoming increasingly stretched with an ever-increasing number of high acuity clinical presentations [6]. In Australia, the most critical (triage category one) patients are always seen immediately [7]. However, in large tertiary teaching hospitals, resuscitation team members may have not previously worked together. In smaller urban and rural departments, exposure to patients in extremis is more infrequent and staffing levels are often minimal. To complicate matters further, available resuscitation protocols are numerous and may contain extraneous detail. Additionally, resuscitation situations are time-critical, and it is not practical for staff to find and digest complicated protocols. Internet access is not always reliable, and locating the correct document often involves the use of passwords during navigation through multiple tabs and links.

Crew Resource Management training was introduced into aviation following the realisation that around 70% of airline crashes involved some degree of failure in human performance [8]. Crew Resources Management makes use of cognitive aids such as checklists to deal with crisis management. Cognitive aids are prompts designed to assist workers in completing a task or series of tasks [2]. A checklist is defined as a ‘type of cognitive aid listing a suggested sequence of actions’ and has been successfully and extensively used in other industries such as aviation [2]. In the USA, anaesthetists subsequently applied some of the key concepts from this ‘non-technical skills training’ to assist with anaesthetic crises [9]. Despite numerous recommendations for the use of checklists in critical care medicine, adoption of such practice has overall been slow [8, 10].

In his book *The Checklist Manifesto*, the American surgeon Dr Gawande highlighted the need for checklists in medical practice [11] to improve patient safety. In 2013, Arriaga and colleagues investigated the use of cognitive aids in intraoperative crises [12]. This US study recruited 17 teams who participated in 106 simulated emergencies. Critical errors decreased by 74% when cognitive aids were used. Six per cent of life-saving steps were missed when cognitive aids were available versus 23% when they were unavailable (*p* < 0.001). Every team performed better when using cognitive aids. This may have been because participants reported feeling better prepared during the emergency scenario. It is also likely that a structured, easy-to-follow approach reduces error rates [12]. This helps to achieve standardisation of care, with the aim of removing unwanted variation.

The design of cognitive aids for use during medical crises continues to evolve. Marshall has suggested that cognitive aids would benefit from more extensive simulation-based usability testing before use [13].

We have crafted a new cognitive aid, the Emergency Protocols Handbook. This handbook is designed to be used at the patient’s bedside during a medical crisis. Aviation experts assisted us with the meticulous graphic design of this cognitive aid. With the potentially stressed reader in mind, font and graphics have been selected to maximise clarity and minimise distraction. The Emergency Protocols Handbook contains 15 adult and 12 paediatric pathways. Current Australian and New Zealand Committee on Resuscitation (ANZCOR) guidelines (www.resus.org.au) were re-formatted into step-by-step pathways, without alteration of any essential content (www.emergencyprotocols.org.au). The handbook was informally tested in simulation scenarios in an emergency room. Repeated iterations (*n* = 20) of the handbook and retesting led to incremental improvements of the various protocols. This study used a trial design similar to that reported in the Arriaga trial [12].

### Objective

This study tested the effectiveness of this cognitive aid in a simulated ER environment by observing team error rates when current resuscitation guidelines were followed, with and without the Emergency Protocols Handbook (www.emergencyprotocols.org.au)

## Methods

### Trial design

The study was a randomised controlled trial. Participants in groups were asked to manage four simulated emergency medicine scenarios: resuscitation of a newborn infant, a 5-year-old with status epilepticus, an adult in cardiac arrest with ventricular tachycardia and an adult who had taken a large overdose of tricyclic antidepressant agents. These scenarios were chosen because they covered the spectrum from neonatal to adult and provided substantial complexity. The tricyclic overdose covered advanced skills and multiple problems (see Appendix for further details).

### Participants and recruitment

**Fig. 1:**
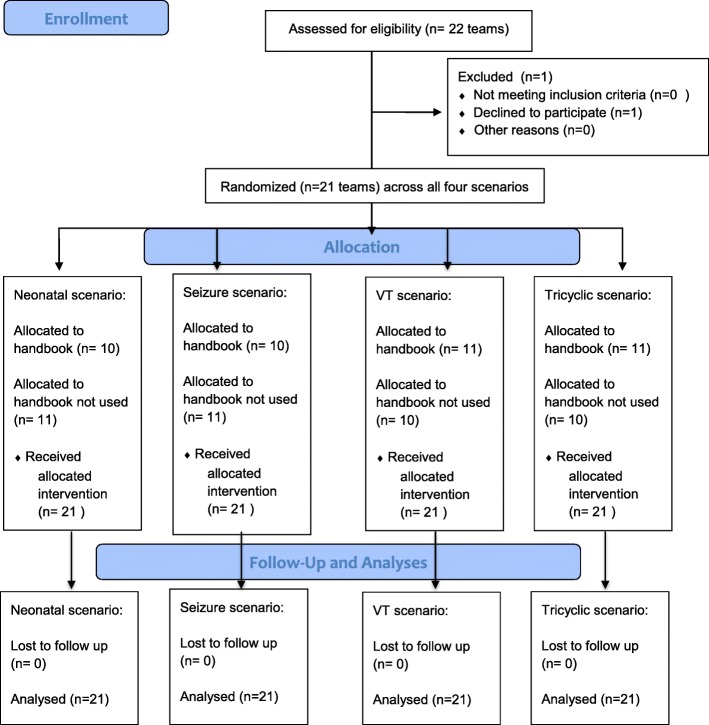
Flowchart

Participants were recruited from public hospitals in Northern New South Wales, Australia, between September 2016 and March 2017 (see Fig. 1). Twenty-one groups were recruited. Eligible participants were qualified nurses or doctors who had worked in an Australian emergency room within the previous 12 months. Students and study personnel were excluded from the trial.

Clinicians from various disciplines attend emergency rooms and thus may find themselves involved in a medical emergency situation. In addition, within Australia, many small and rural emergency rooms have only a single doctor and nurse on call or on duty. During a serious medical emergency, these departments enlist the additional help of any readily accessible staff who have medical training such as ward nurses, paramedics or the nearest local general practitioner. Therefore, the study investigators sought to reflect this diversity of medical provider by recruiting a small number of non-emergency room nurses and doctors into the trial.

Participants were enrolled by means of their reply to an emailed flyer regarding the trial or following a verbal request from either the researchers, managers or education staff within the hospitals. All participants received a written information statement prior to consenting to take part in the trial. Ethics approval was obtained from the Northern New South Wales Local Health District Human Research Ethics Committee (LNR138). Participants gave written informed consent. Participants enrolled as individuals and not as a team.

### Random allocation

Groups were randomised to complete two simulated emergencies with the use of the Emergency Protocols Handbook and two simulations without the handbook. Using SPSS v22 (IBM Corp.), a biostatistician (MR) generated four different randomisation sequences. Blocks of six were used to allocate teams to scenarios. The randomisation process was chosen to ensure balance, reduce bias involving scenario and handbook use, and to allow all groups the educational benefit of performing all four scenarios. Administrative staff, with no clinical involvement in the trial, blindly selected one sequence. Hence, the random allocator was blinded to the group allocation. The same administration staff then prepared sequentially numbered, opaque envelopes (group randomisation allocation), enclosing the list of scenarios (identified by their number only) and whether they were to perform the scenario with or without the handbook. Participants available for each session were grouped by an investigator (CH, DR) to ensure that there was a combination of doctors and nurses in each group prior to randomisation allocation. After the individuals provided consent, a simulation laboratory staff member assisting in the trial assigned the envelopes in numerical sequence to the groups. The staff member was also blinded to group allocation to prevent selection bias. The envelope was opened by an investigator (CH, DR, SP) just prior to the commencement of the scenario.

The groups had the handbooks for two of the four scenarios but in a random order. Although it is theoretically possible that a participant could have studied the book to use in other scenarios, we did not observe this behaviour and there was no time to do this between scenarios. Indeed, the groups were under time pressure and appeared to be concentrating only on the scenario in front of them. There are dozens of protocols in the handbook and the possibility of a group member selecting the correct protocol to study was considered to be small.

The groups were unaware of, and thus blinded to, the outcome measures being assessed, but the investigator in the room was aware. Data analysts were not blinded to the group allocation.

### Intervention

The Emergency Protocols Handbook has been designed, in consultation with aviation experts, as a cognitive aid for clinical staff who have to manage medical emergencies that are both life-threatening and time-critical. Current protocols were reformatted as simple step-by-step pathways designed to be read out loud during a resuscitation event. There are no flow diagrams and minimal explanatory text. Longer stepwise protocols extend over several pages. The content of the protocols was in accordance with the current Australian and New Zealand Committee on Resuscitation (ANZCOR) guidelines and not altered. The protocols address immediate resuscitation management and in some cases guide disposition (e.g. to a paediatric intensive care unit).

The intervention was conducted with manikins in a high-fidelity simulation laboratory at the University Centre for Rural Health, Lismore, New South Wales, Australia. The rooms were set up as medium-sized resuscitation rooms, with appropriate lighting, full monitoring equipment, defibrillator, adult and/or paediatric airway trolley, medication trolley, neonatal resuscitation cot with resuscitation equipment, whiteboard, telephone, four video cameras and a separate microphone. Participants were provided with the handbook and briefly familiarised with the step-by-step nature of the pathways by two investigators (CH, DR). They were given no further instructions on how to use it within their group setting. A maximum time allocation of 15 min was allowed for each simulation. The teams were given an initial short verbal prompt and then asked to manage the situation. If teams had not finished the scenario in 15 min, then any of the 15 key tasks not yet completed were instantly considered as not completed. However, all teams completed the simulations within 15 min.

The response of the groups was recorded on digital media and analysed for 15 primary outcome measures for each scenario.

Prior to the commencement of the study, the cognitive aid was available online but was not part of the standardised usual care. No participant had received any formal training regarding the correct use of this cognitive aid prior to the trial. Participants were unaware of the medical content of the simulations before the study. Each group was told that they would have to manage four medical emergencies and that the overall performance of the group would be evaluated.

One investigator (CH, DR) stayed in the room with each group to give previously scripted verbal prompts in response to the actions of the group. The simulations were performed in a consecutive manner, but the order in which the scenarios were performed varied between the groups.

### Usual care

Without the handbook, clinicians in the teams relied on existing printed Australian New Zealand Committee on Resuscitation algorithms and memory of usual care. All groups were given access to standard ANZCOR resuscitation guidelines and were permitted to use computers and phones to access information.

### Outcomes

The primary outcome measure was error rates, calculated as the total number of errors made out of 15 key tasks per scenario, see the list in Appendix. The 15 key tasks per scenario were chosen to mirror current resuscitation algorithms and represent important life-preserving steps. Completion of key tasks in order of sequence was important. Errors included omission, incorrect sequences, incorrect drugs, wrong settings or missed steps. The error rate is presented as percentages or proportions for each scenario/handbook combination. Thus, there were a total of 60 (15 × 4 scenarios) key tasks for each group as each group performed four scenarios. One investigator (DR) viewed and rated all recordings, and two investigators (CH, SP) rated and viewed a subset of the recordings to score each key task. The inter-rater reliability was assessed using a subset of the scenarios (see Appendix for details).

The secondary outcome was the participant’s subjective experiences of the trial. Participants were asked to fill out a questionnaire to evaluate their subjective experience of using the handbook. They were asked to indicate their agreement with 12 statements, using a 5-point Likert scale (see Table 4). The questionnaire was filled out at the end of the four scenarios.

### Statistical analyses

Statistical analysis was performed using SPSS Version22 (SPSS IBM, New York, USA). The error rates (number of errors/15) per scenario were compared with and without the handbook, while accounting for group dependence. One point was allocated for each correct key process resulting in a possible range of scores between 0 and 15. As expected, total error scores followed a Poisson distribution for scenarios that did and did not use the handbook. A generalised mixed model in the form of a binomial model and logit link was fitted on the proportion of errors with each ‘scenario’ and ‘handbook use’ (within subject or repeated measure) nested in ‘group’ (subject level variable). Main effects were calculated for the handbook (used versus not used) and scenario (neonatal  resuscitation, paediatric seizures, adult pulseless ventricular tachycardia (VT), tricyclic overdose) and the interaction effect between handbook use and scenario. Several covariance structures were investigated to explore the repeated measures of the four scenarios within each group including diagonal, compound symmetry, unstructured and identity. The model with the lowest Akaike Information Criteria (AIC) measure of fit was considered to best represent the data.

## Results

### Sample description

Seventy-five participants (38 doctors and 37 nurses) participated in 21 groups. The groups were exposed to 84 simulated crises, giving a total of 1260 key tasks. Group size ranged between three and six team members. The majority of groups (*n* = 13) consisted of three members, followed by five groups of four participants, two groups of five participants and one group of six participants. The average group size was 3.6. All groups had at least one doctor. Doctors made up 51% of the group composition, and this ranged from 25 to 100%. Males were on average 41% of the group makeup, ranging from 0 to 75%. The characteristics of the participants are shown in Table 1. The inter-rater reliability testing is presented in Appendix. All rater pairs indicated moderate or substantial agreements with respect to kappa scores (0.56, 0.64, 0.67) and good or excellent intra-class correlation coefficient scores (0.72, 0.82, 0.87) as well as percentage agreements in excess of 80% (83.4%, 85.7%, 96.7%).

**Table 1:** Baseline characteristics of participants (*N* = 75)

		Number	Percentage
Gender	Female	45	60
	Male	30	40
Discipline	Medical	38	51
	Nursing	37	49
Medical (*n* = 35)^1^	Junior Medical Officer	20	57
	Resident Medical Officer	1	3
	Registrar	7	20
	GP/GP VMO	4	11
	Career Medical Officer	1	3
	Staff specialist/VMO	2	6
Nursing (*n* = 35)^2^	Registered nurse	23	66
	Nursing manager	3	9
	Clinical nurse specialist/educator	8	23
	Nurse practitioner	1	3
Hospital type^3^	Base and tertiary	44	61
	Rural	22	31
	Rural and base	1	1
	Tertiary	3	4
	None	2	3

^1^Missing data, *n* = 3

^2^Missing data, *n* = 2

^3^Missing data, *n* = 3

### Analyses of handbook use

The analysis was by original assigned groups. Table 2 presents the absolute number of errors and descriptive data on error rates (as a percentage) for scenario and handbook use. The handbook was used in 43 scenarios and not used in 41 scenarios.

**Table 2:** Descriptive statistics for error rates by scenario and handbook use

Scenario	Handbook	Number of errors	Total possible errors	Mean error rate (%)	SE of mean error rate	Number of teams	Median error rate (%)	Error rate range (%)
Neonatal	Not used	56	150	37.33	4.98	10	40.0	13.3–60.0
	Used	24	165	14.54	3.21	11	13.3	0–33.3
	Total	80	315	25.39	3.81	21	20.0	0–60.0
Seizures	Not used	55	165	33.33	4.11	11	40.0	6.7–53.3
	Used	18	150	12.00	2.39	10	13.3	0–26.7
	Total	73	315	23.17	3.36	21	20.0	0–53.3
Tricyclic	Not used	66	150	44.00	7.77	10	50.0	6.7–73.3
	Used	33	165	20.00	4.21	11	26.7	0–40.0
	Total	99	315	31.42	4.97	21	26.7	0–73.3
VT	Not used	62	150	41.33	3.82	10	40.0	13.3–60.0
	Used	46	165	27.87	2.95	11	33.3	13.3–40.0
	Total	108	315	34.28	2.77	21	40.0	13.3–60.0
Total	Not used	239	615	38.86	2.65	41	40.0	6.7–73.3
	Used	121	645	18.75	1.84	43	20.0	0–40.0
	Total	360	1260	28.57	1.93	84	26.7	0–73.3

The results of the binomial generalised mixed model analysis showed statistically significant main effects of handbook use (*F*_1, 64_ = 42.8, *p* < 0.001) and scenario (*F*_3, 35_ = 5.27, *p* = 0.004), but the interaction (handbook use by scenario) was not significant (*F*_3, 35_ = 1.65, *p* = 0.197). The diagonal covariance structure displayed the best goodness of fit for the model (AIC = 189.327), suggesting heterogeneous variances and zero covariances.

**Fig. 2:**
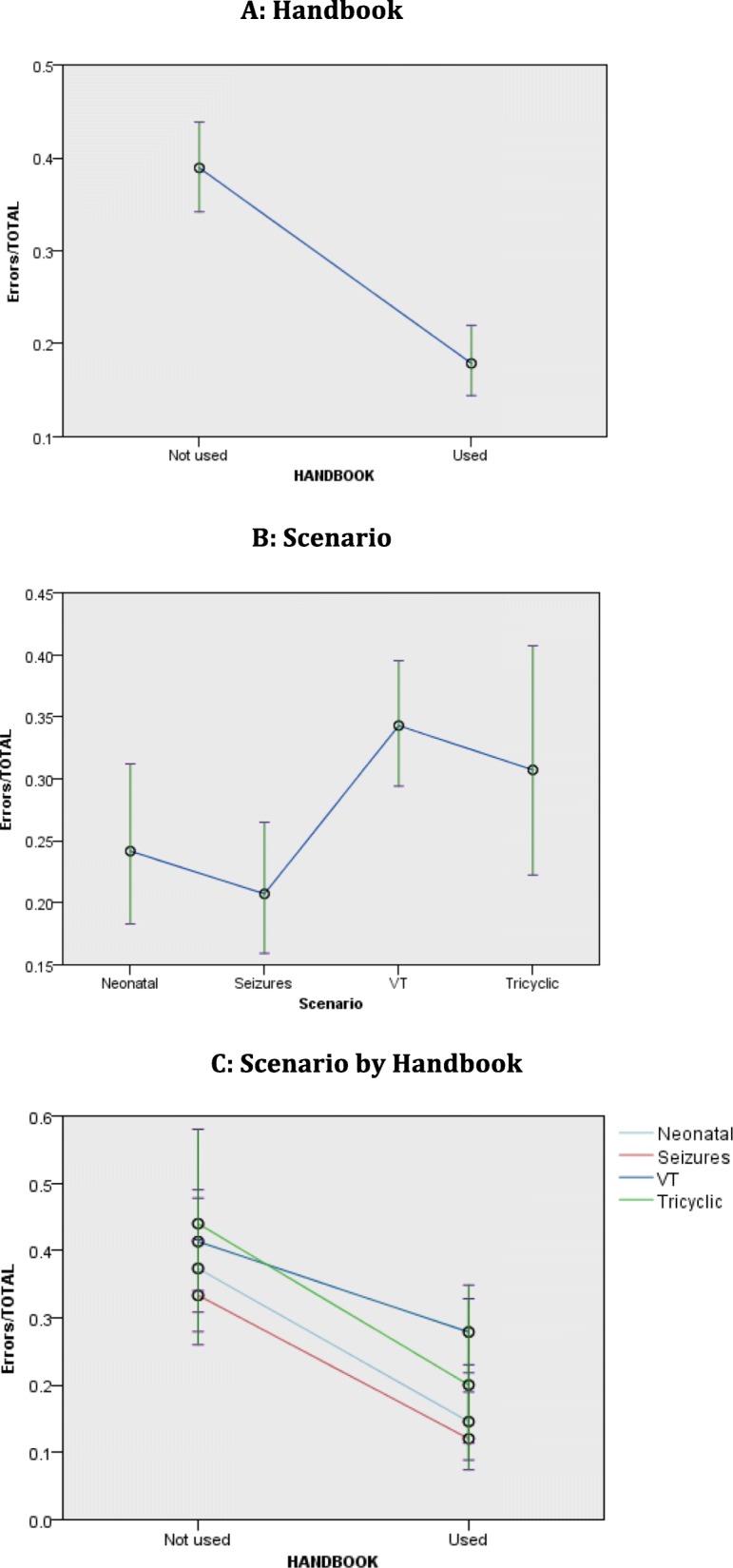
Estimated means and 95% confidence intervals for error rates for handbook use (**a**), scenario (**b**) and handbook by scenarios interaction (**c**)

Overall, scenarios using the handbook exhibited significantly lower estimated error rates: 17.9% (95% CI 14.4–22.0) versus 38.9% (95% CI 34.2–43.9), for a relative reduction in error of 54.0% (95% CI 49.9–57.9) (Table 3, Fig. 2a). The absolute risk reduction (ARR) rate is 38.9–17.9% = 21%. Table 3 presents the estimated means and 95% confidence intervals for error rate proportions for the main effects of scenario and handbook use as well as their interaction derived from the generalised mixed model analyses together with significant pairwise contrasts.

**Table 3:** Estimated error rate proportion means and 95% confidence intervals (CI) of the scenario and handbook main effects and interaction with significant pairwise contrasts and *p* value

	Scenario	Number	Handbook	Error rate proportion	SE	95% CI	Significant pairwise contrasts		*p* value
Main		41	Not used	0.389	0.024	0.342–0.439			
		43	Used	0.179	0.019	0.144–0.220		0.210	<0.001
Main	Neonatal	21		0.242	0.031	0.183–0.312	N-VT	0.101	0.014
	Seizures	21		0.207	0.025	0.159–0.265	VT-Seiz	0.136	<0.001
	VT	21		0.343	0.024	0.294–0.395			
	Tricyclic	21		0.307	0.045	0.222–0.407			
Interaction	Neonatal	10	Not used	0.373	0.048	0.279–0.478			
	Seizures	11	Not used	0.333	0.038	0.260–0.416			
	VT	10	Not used	0.413	0.036	0.340–0.490			
	Tricyclic	10	Not used	0.440	0.067	0.308–0.581			
	Neonatal	11	Used	0.145	0.033	0.088–0.230	Used	0.228	0.001
	Seizures	10	Used	0.120	0.027	0.074–0.189	Versus not used	0.213	<0.001
	VT	11	Used	0.279	0.031	0.218–0.349	Not	0.135	0.011
	Tricyclic	11	Used	0.200	0.051	0.113–0.328	Used	0.240	0.010

As there was no significant interaction between handbook use and scenario, the pattern of error rates was similar between the two levels of handbook use for the four scenarios. Handbook use always provided significantly lower error rates irrespective of scenario (see Table 3 and Fig. 2c). For the individual scenarios, the reduction in error rates, between no handbook and handbook, were Neonatal 61.1%, Seizures 63.9%, VT 32.4% and Tricyclic overdose 54.5%. The VT scenario had the highest error rate followed by the Tricyclic scenario. The Tricyclic scenario without the handbook also showed a greater variation in the error rate.

### Process measures

Despite general high agreement rates with the different aspects related to the handbook, about one in three participants were neutral or disagreed with the statement ‘I prefer linear steps to flowcharts.’ (Table 4). However, almost all participants would use the handbook in real life (97%), and 93% would want the handbook to be used if they or a family member were to experience a medical emergency.

**Table 4:** Process measures

	Number	Percentage	Number	Percentage	Number	Percentage	Number	Percentage	Number	Percentage
	Strongly disagree		Disagree		Neutral		Agree		Strongly agree	
Sessions										
The sessions were realistic	0	0.0	1	1.4	1	1.4	39	54.2	31	43.1
The sessions were challenging	0	0.0	0	0.0	1	1.4	30	41.7	41	56.9
The sessions prompted realistic responses from me	0	0.0	1	1.4	8	12.5	31	43.1	31	43.1
The sessions should be taken by all emergency staff	0	0.0	0	0.0	1	1.4	25	34.7	46	63.9
Protocol										
The Protocol handbook was easy to use	0	0.0	0	0.0	3	3.9	35	46.1	38	50.0
The font was clear and easy to read	0	0.0	0	0.0	1	1.3	24	31.6	51	67.1
I prefer linear steps to flowcharts	1	1.3	2	2.6	21	27.6	25	32.9	27	35.5
The Protocol handbook helped me to manage these emergencies	0	0.0	0	0.0	1	1.3	29	38.7	45	60.0
I would use the Protocol handbook if presented with these emergencies in real life	0	0.0	0	0.0	2	2.6	17	22.4	57	75.0
If I, or a family member, experienced these emergencies, I would want the Protocol handbook used	0	0.0	0	0.0	5	6.6	20	26.3	51	67.1
Overall quality	Unacceptable		Poor		Fair		Good		Excellent	
Sessions	0	0.0	0	0.0	0	0.0	15	20.8	57	79.2
Protocol handbook	0	0.0	0	0.0	0	0.0	14	18.4	61	80.3

## Discussion

### Overview of findings

Resuscitation teams may experience cognitive overload, stress and incomplete recall of previous training during resuscitation events in the emergency room [1]. In this simulation-based study, we found that the use of a cognitive aid led to a significant reduction in error rates. All the groups in the trial reduced their error rates by at least 20% when they were given access to this Emergency Protocols Handbook. Overall, there was a 54% reduction in errors made across all four scenarios.

After participation in the trial, almost all participants (97%) agreed that they would want to use this cognitive aid, if faced with a real medical crisis situation.

The trial results show that without the handbook, all teams, regardless of configuration and experience level, were less likely to follow recommended guidelines and more likely to make errors in key steps of management during time-critical minutes.

### Value of cognitive aids

Operating rooms and emergency rooms are high-stakes environments where patient safety is at risk from errors of judgement or management. In 2013, Arriaga and team [12] demonstrated that checklist use during simulated operating room crises resulted in a nearly 75% reduction in failure to adhere to critical steps in management. Our research adds to this finding by demonstrating a large (54%) reduction in errors rates in key tasks when a cognitive aid is used to manage medical crises within a simulated emergency room. Marshall identified knowledge gaps in cognitive aid research and argues that manikin-based simulation is an ideal method to trial cognitive aids [13]. Similar to medical devices, high-quality cognitive aids depend on the right content and design and are supported by appropriate training to enable task execution (usability) [2]. Marshall concluded in a systematic literature review that most studies focused on the right content (e.g. national guidelines) and less on design processes (such as iterative processes), presentation and usability. Our handbook was based on current guidelines. Moreover, there is some legitimacy offered for our handbook as it has already been endorsed by the Australian Agency for Clinical Innovation, the Australian Emergency Care Institute (ECI) and the Australian College of Rural and Remote Medicine. Furthermore, our handbook was initially developed with aviation experts to refine graphic design and ease of reading given their expertise in this area. Lastly, there is evidence to support the linear design [14]. A 2016 multicentre study amongst anaesthetists assessed how contrasting designs of cognitive aids affected team performance during simulated intraoperative anaphylaxis crises. The teams (*n* = 24) were randomly assigned to a counterbalanced order of no cognitive aid, a linear cognitive aid or a branched cognitive aid. Team functioning scores were significantly higher with a linear designed cognitive aid when compared to a branched version of the cognitive aid or no cognitive aid.

Despite the high acceptance rates, about one in three participants, were neutral or disagreed with the statement ‘I prefer linear steps to flowcharts’. It may be that clinicians are used to working with flowcharts, and changing their way of working may require some time to get used to, or alternatively that flowcharts with linear steps do not suit all clinicians.

### Scenario-specific challenges

The reduction in error rate was lowest in the VT group. The causes for this are uncertain, although reliance on memory, at the expense of direct reading from the pages of the handbook, may have contributed. Of all the scenarios, VT is the only one that participants had previously been exposed to as part of their regular mandatory training requirements. Certification in advanced life support is a requirement for clinical staff in Australian emergency rooms. Staff members designated to respond to collapsed persons receive training in resuscitation strategies (Australasian College for Emergency Medicine-Policy on Early Access to Defibrillation for Cardiac Arrest), which includes VT. Of the four scenarios, VT is the one most likely to have been previously committed to memory. We postulate that reliance on knowledge already committed to memory led to errors in key tasks in both groups. Participants likely did not follow the step-by-step approach in the handbook. Their previous training did not prevent errors in key tasks. It is surprising that the highest error rate was in the scenario where clinicians are most commonly drilled (VT). We checked whether there was a pattern across the errors made or whether there were one or two common errors made across the board in this scenario, e.g. failure to connect capnography. There was no pattern in the VT errors made. We also postulate that the higher error rates may have been due to the format of the handbook. Some groups commented that the handbook was more difficult to navigate during this scenario, as multiple pages had to be consulted. Groups had only received minimal training in the use of the handbook by reviewing the page of steps to be followed for an emergency scenario that was not included in the trial. We had not given a full explanation of how to navigate multiple pages simultaneously in the handbook. There may therefore have been some issues with the current format of the VT scenario. We will continue to strive for simplicity of presentation in the handbook and will re-evaluate how we present the VT scenario in future editions. The greatest reduction in error was in the tricyclic overdose scenario. Again, the cause for this is uncertain. This is a relatively rare medical crisis, requiring special management. It is possible that participants were aware of their lack of stored memory for this emergency situation and were thus more willing to adhere to the steps in the handbook, if given the opportunity.

It could be argued that positive results of the handbook might decline with more experienced emergency doctors and/or less critical ER scenarios. This might explain the huge effect in the tricyclic overdose scenario (which is challenging and rare). Another explanation for the large effect may be that the handbook could be more effective in rural settings where resuscitation scenarios are less common, so rural staff might be more likely to follow the handbook compared with experienced urban clinicians. This could be the case because rural clinicians are less frequently exposed to the scenarios and therefore may not have the knowledge readily available in their memory. The strong results in the tricyclic overdose scenario, hypothesised to be due to its rarity, support this idea. Further research could examine who benefits most from a cognitive aid. On the other hand, the VT scenario findings potentially suggest that reliance on memory alone is inaccurate regardless of the participant’s level of expertise.

### Study limitations

Our study contributes to cognitive aid research in a simulated environment. However, it is unknown whether interventions tested by simulation will perform in a similar manner during real clinical situations. In such real life emergency situations it would be difficult to conduct randomised trials so high-fidelity simulation laboratories play an essential part in reducing error. There is a support that simulations are meaningful for real-world situations [15, 16]. Simulations allow clinicians to develop and practice non-technical skills in a safe and controlled environment without comprising patient safety [17]. In a real clinical situation, team composition and other factors may change that cannot easily be simulated. Our trial did not account for rural versus urban settings. Busy urban centres might have different responses to the implementation of the cognitive aid than rural centres. Differences in urban versus rural setting have not yet been thoroughly investigated and further trial research is warranted.

Another study limitation was team composition. Firstly, there were more junior doctors than senior doctors and more senior nurses than junior nurses. There was at least one doctor in every group. Selection bias may have occurred due to the researchers recruiting a small number of non-emergency room nurses and doctors into the trial, although this was conducted to reflect diversity of emergency medical care teams. This also reflects practice in rural areas. Another limitation was that the individuals who were assessing the video recordings of the scenarios were not blinded to whether the handbook was used as this was not possible. This may have positively influenced the assessment of the trial; however, we tried to maximise objectivity by having two reviewers independently assessing each scenario. Secondly, the groups ranged in size from three to six participants. This may have influenced the results. However, the results consistently showed across group size a reduction in error rates when the handbook was used. Another limitation was that one of the investigators was in the simulation room and interacting with the participants during the scenario, which potentially introduced a source of bias that cannot be removed. However, the observing investigator sat in the corner of the room and was only allowed to deliver pre-scripted responses such as ‘the retrieval team are delayed’ if prompted by the participants. Video recording shows that the observing investigator’s participation was indeed very minimal but we are not able to exclude this bias, and this should be taken into account when interpreting the results. Another limitation was that the effect of the scenario sequence was not fully counterbalanced.

### Future work

It may be that familiarity and dexterity with this cognitive aid may increase with further practice and training. However, it may also lead to the opposite effect given that in this trial, the scenario where participants had the most experience (VT) was the scenario that teams performed the worst in, with or without the handbook. This suggests that longitudinal research with the handbook may be required to assess whether extended familiarity and use of the handbook in real clinical settings is actually sustained. It is possible that extended familiarity with the handbook could cause a similar effect to that of participants receiving additional VT training (e.g. a degradation in completion of key tasks).

Future studies on linear versus branched cognitive aids are required to test why one third of the participants did not favour linear steps. It could be that resuscitation algorithms are commonly published and taught with branching and looping steps, making users unfamiliar and uncomfortable with linear steps. Familiarity with linear steps could change this perception. Digital technologies can also increase familiarity with linear steps, or allow for branched cognitive aids, as suggested by Marshall and colleagues [14]. Electronic cognitive aids might also address some of the implementation challenges mentioned above, such as how do participants recall which patient conditions are covered by the handbook; charting patient vitals and history into a rapid triage screen could automatically pull up the relevant prompts for users without their need to consider competing care pathways. An application for mobile phones, based on the handbook, is under development and warrants further research. However, electronic cognitive aids have multiple potential failure points (e.g. passwords, network access, charging and compatibility issues) that paper-based solutions avoid.

The demonstration of significant benefit to the patient from the use of a cognitive aid, combined with evidence from other industries, suggests that the next step is to implement cognitive aids in practice [12]. Optimal resuscitation care is achieved through standardisation. Standardised practice results in less unwanted variation, thereby reducing errors and improving outcomes. However, the challenge is to promulgate standardised processes. Implementation strategies will be required to change practice. Questions will need to be further investigated to develop implementation strategies for the handbook use, such as how staff will use the handbook, why they will use it, what barriers exist and what will enable its use in clinical practice. Further research could examine the implementation in urban and rural settings. A multitude of factors affects cognitive aid implementation, including social behaviour. No team naturally designated someone to read the handbook aloud. Indeed, training in the use of the handbook will be crucial for widespread adoption [2], including the need for a ‘Resuscitation Guide Manager’. The latter could potentially be a nursing role or someone with expertise in human factor sciences. Dedicating an individual to this role would conceivably change who is accountable for the tasks and change patterns of task delegation and who feels responsible for what. Therefore, training will be paramount prior to implementation in live clinical settings, and further work needs to be conducted in this area. Prior to implementation of the handbook, staff will need to be trained in where the handbook would be placed and how clinicians will be cued to use the handbook. Staff will also need to be trained in the contents of the handbook so they remember whether or not the protocol for the patient’s condition is in the handbook. It is anticipated that the handbook will be kept in a prominent location in the resuscitation room for ease of access when required. Even though the handbook format was designed to be read aloud during a resuscitation event, our participants were not instructed to do this. With additional group training and practice with the handbook, we predict that error rates might fall even further—and this would be an interesting future study. Future work should be completed to answer questions regarding implementation success, using pilot studies in live clinical environments.

In the next decade, human performance training for teams may potentially transform how medical care is delivered [18]. Healthcare team workers will learn how to enhance their non-technical skills, enabling them to improve their own conduct within a team setting. Behaviours can be changed [18]. We predict that bedside cognitive aids and high-order team skills will become expected practice, and errors will be decreased. The next steps will involve the further development of innovative human performance team training methods that are evaluated in both simulated and real clinical settings. Although it may take years to measure and determine whether such human performance team training translates into safer patient care, it seems prudent to become early adopters of this practice [12]. It also seems intuitive that a more structured and consistent approach to resuscitation will lessen clinician stress and lead to improved outcomes for our patients. Further research in this area is warranted.

## Conclusion

Our study showed that by following the step-by-step linear pathways in the Emergency Protocols Handbook, clinicians more than halved their teams’ rate of error, across four simulated medical crises. This is important because the handbook improves human performance in team settings and can reduce harm for patients. It is expected that in the future, health care team workers will learn how to enhance their non-technical skills, enabling them to improve their own conduct within a team setting.

## Data Availability

The datasets generated and/or analysed during the current study are not publicly available due to the data being used for specified purposes within the ethics approval.

## Appendix

### Sample size calculation

Because we were unable to find background data on the performance during emergency room crises, we followed a similar process that was used in the Simulation-Based Trial of Surgical-Crisis Checklists [12]. In Arriaga and colleagues’ trial, the rates of failure of adherence to best practices were expected on average to be 15% without the checklist and 9% with the checklist. We used similar parameters although we were more conservative, so in our trial, the rates of failure of adherence to best practices were expected on average to be 15% without the Handbook of Emergency Protocols and 10% with the Handbook.

With a two-sided type I error rate of 5%, we estimated the trial to have over 80% power to detect a decrease in failure rate from 15 to 10% with the Handbook with 20 teams and 683 total process measures with the Handbook and 683 without. In order to adjust for the repeated measures structure, we assumed an intra-cluster correlation coefficient (ICC) within teams to be approximately 0.05, with a design effect of 1.15, leading to a final sample of 593 total process measures with the Handbook and without. Twenty teams were included leading to 1200 process in total (600 with the Handbook and 600 without the Handbook).

### Primary outcome measures

#### Scenario 1: Paediatric seizures

‘This is a 5-year-old girl brought in by her teacher. She was well then started fitting 5 min ago.

1. Call for help

2. Open airway

3. Give oxygen

4. Request appropriate bloods (FBC, EUC, Ca Mg Ph, anticonvulsant levels, culture)

5. Check blood glucose

‘Blood glucose is 2.6’

6. Give IV dextrose 10%

7. Give 2 mL/kg of IV dextrose 10%

8. Give correct first benzodiazepine (midazolam or diazepam)

9. Give a correct dose of first benzodiazepine

‘Five minutes has passed, the patient continues to fit’

10. Give repeat dose of benzodiazepine

“Five minutes has passed, the patient continues to fit”

11. Give correct third-line drug (phenytoin or levetiracetam or phenobarbitone)

12. Give a correct dose of third-line drug

‘Seizures continue’

13. Monitor ECG, oxygen saturations and respiratory rate

14. Call for expert advice

15. Consider RSI

#### Scenario 2: Adult Pulseless Ventricular Tachycardia

‘This is a 60-year-old male who has suddenly stopped talking and turned pale’

1. Check for output

2. Start CPR within 1 min

3. Call for help

4. Attach monitor

5. Give oxygen

6. Attempt IV access

‘IV access is successful’

7. Request or place waveform capnography

8. Give shock within 2 min

9. Give 200 J for shock

10. Consider four Hs (hypoxia, hypovolaemia, hypothermia/hyperthermia, hyperkalaemia/hypokalaemia/metabolic disorder)

11. Consider four Ts (thrombosis pulmonary/coronary, tension pneumothorax, toxins, tamponade)

12. Give IV adrenaline 1 mg after the second shock

13. Give IV amiodarone 300 mg after the third shock

‘The patient has a potassium of 9’

14. Give IV calcium (calcium gluconate 10% 20 mL or calcium chloride 10% 10 mL)

15. Give IV insulin 10 units and IV glucose 50% 50 mL

#### Scenario 3: Newborn resuscitation

‘This is a precipitous delivery in ED at 34 weeks, the baby is floppy with no respiratory effort’

1. Call for help

2. Attempt or request warming

3. Open airway

4. Give stimulation

5. Check heart rate

‘Heart rate is 80’

6. Give positive pressure ventilation

7. Check oxygen saturations on the right hand

8. Recheck airway

‘Heart rate is 50’

9. Start CPR within 10 s

10. Give CPR in a ratio of three compressions then a breath

11. Give 100% oxygen

12. Insert LMA or intubate

13. Gain IV or IO access

14. Give IV/IO adrenaline 1:10 000 0.25 mL

15. Give IV/IO normal saline 20 mL

#### Scenario 4: Adult tricyclic overdose

‘The patient is a 30-year-old male whose girlfriend left him today. He is talking and agitated. Here are his medications’ (bag includes an empty box of tricyclic antidepressant)

1. Identify likely toxin (tricyclic antidepressant)

2. Attempt IV access

‘IV access is successful’

3. Check blood glucose

4. Request paracetamol level

5. Request ECG

‘Here is the ECG’

6. Identify R wave > 3 mm in aVR

7. Identify QRS > 100

‘Patient is having a seizure’

8. Give benzodiazepine (midazolam or diazepam)

9. Give IV sodium bicarbonate

10. Give 1 to 2 mL/kg of IV sodium bicarbonate 8.4%

‘The nurse asks if she can give the patient some phenytoin’

11. Refuse phenytoin

‘The patient has gone into VF’

12. Start CPR within 1 min

13. Give IV sodium bicarbonate

14. Give 1 to 2 mL/kg of IV sodium bicarbonate 8.4%

15. Give a repeat dose of IV sodium bicarbonate

### Inter-rater reliability video analyses

Rater 1 (DR) marked all groups for the analyses. Rater 2 (SP) marked the first four groups. Rater 3 (AX) marked a random 15% of groups. Rater 1 and rater 4 (CH) marked a pilot group together and the first 18 scenarios. DR and CH coded the pilot group independently then compared their ratings and discussed discrepancies and their reasons for their rating and reached consensus. They repeated this for the first 18 scenarios.

### Statistical analyses

Inter-rater reliability was assessed by using intra-class correlation (ICC) for the error rate for each scenario and the Kappa coefficient and percentage agreement with the 15 outcome (binary) measures for each scenario**.** Commonly cited cut-offs for qualitative ratings of agreement based on ICC values are inter-rater reliability (IRR) being ‘poor’ for ICC values less than .40, ‘fair’ for values between .40 and .59, ‘good’ for values between .60 and .74 and ‘excellent’ for values above .75. The following Kappa ranges indicate an assessment of agreement to be ‘substantial’ if higher than 0.60, ‘moderate’ for 0.41–0.60 and ‘low or poor’ if less than 0.40.

## Results

All rater pairs indicated moderate or substantial agreements with respect to kappa scores (0.56, 064, 0.67) and good or excellent ICC scores (0.72, 0.82, 0.87) as well as percentage agreements for individual items in excess of 80% (83.4%, 85.7%, 96.7%) (Appendix in Table 5).

**Table 5:** Rater pairs indicated moderate or substantial agreements with respect to kappa scores and good or excellent ICC scores as well as percentage agreements for individual items

Rater	Number of scenarios rated	Intraclass correlation (ICC)	Kappa	Percentage agreement
1, 2	16	0.716 (0.369–0.890)	0.564	83.4
1, 3	13	0.816 (0.515–0.939)	0.644	85.7
1, 4	18	0.870 (0.692–0.949)	0.667	96.7

## Abbreviations

AIC: Akaike Information Criterion
CI: Confidence interval
DF: Degrees of freedom
ER: Emergency room
GP: General practitioner
ICC: Intra-class correlation
IRR: Inter-rater reliability
VMO: Visiting Medical Officer
VT: Adult Pulseless Ventricular Tachycardia

## References

[1] Schull MJ, Ferris LE (2001). Tu et al. Problems for clinical judgement: 3. Thinking clearly in an emergency. CMAJ.

[2] Marshall S (2013). The use of cognitive aids during emergencies in anesthesia: a review of the literature. Anesth Analg.

[3] Roth E, Mumaw R, Lewis P. An empirical investigation of operator performance in cognitively demanding simulated emergencies. Nuclear Regulatory Commission, Washington, DC (United States). Div. of Systems Research; Westinghouse Electric Corp., Pittsburgh, PA (United States). Science and Technology Center, 1994.

[4] Kuhlmann S, Piel M, Wolf OT (2005). Impaired memory retrieval after psychosocial stress in healthy young men. Journal Neurosci.

[5] Hales BM, Pronovost PJ (2006). The checklist—a tool for error management and performance improvement. J Crit Care.

[6] Forero R, Nugus P. Australasian College for Emergency Medicine (ACEM) literature review on the Australasian Triage Scale (ATS). Institute of Health Innovation, 2012.

[7] Australian Institute of Health and Welfare (AIHW). Emergency department care 2015–16: Australian hospital statistics. Health services series no. 72. Cat. no. HSE 182. Canberra: AIHW, 2016.

[8] Helmreich RL (2000). On error management: lessons from aviation. BMJ.

[9] Neily J, DeRosier JM, Mills PD (2007). Awareness and use of a cognitive aid for anesthesiology. The Joint Commission Journal on Quality and Patient Safety.

[10] Kohn LT, Corrigan JM, Donaldson MS. To err is human: building a safer health system: Washington (DC): National Academies Press, 2000. 25077248

[11] Gawande A (2010). The checklist manifesto: how to get things right.

[12] Arriaga AF, Bader AM, Wong JM (2013). Simulation-based trial of surgical-crisis checklists. N Engl J Med.

[13] Marshall SD (2017). Helping experts and expert teams perform under duress: an agenda for cognitive aid research. Anaesthesia.

[14] Marshall SD, Sanderson P, Mcintosh CA, Kolawole H (2016). The effect of two cognitive aid designs on team functioning during intra-operative anaphylaxis emergencies: a multi-centre simulation study. Anaesthesia..

[15] Weller J, Henderson R, Webster CS, Shulruf B, Torrie J, Davies E, Henderson K, Frampton C, Merry AF (2014). Building the evidence on simulation validity comparison of anesthesiologists’ communication patterns in real and simulated cases. Anesthesiology..

[16] Merry AF, Hannam JA, Webster CS, Edwards KE, Torrie J, Frampton C, Wheeler DW, Gupta AK, Mahajan RP, Evley R, Weller JM (2017). Retesting the hypothesis of a clinical randomized controlled trial in a simulation environment to validate anesthesia simulation in error research (the VASER Study). Anesthesiology..

[17] Lewis R, Strachan A, Mckenzie Smith M (2012). Is high fidelity simulation the most effective method for the development of non-technical skills in nursing? A review of the current evidence. Open Nurs J..

[18] Hamman WR, Beaubien JM, Beaudin-Seiler BM (2009). Simulation for the training of human performance and technical skills: the intersection of how we will train health care professionals in the future. J Grad Med Educ..

